# Diversity in Polygenic Risk of Primary Open-Angle Glaucoma

**DOI:** 10.3390/genes14010111

**Published:** 2022-12-30

**Authors:** Jessica N. Cooke Bailey, Kaitlyn L. Funk, Lauren A. Cruz, Andrea R. Waksmunski, Tyler G. Kinzy, Janey L. Wiggs, Michael A. Hauser

**Affiliations:** 1 Cleveland Institute for Computational Biology, Case Western Reserve University School of Medicine, Cleveland, OH 44106, USA; 2 Department of Population and Quantitative Health Sciences, Case Western Reserve University School of Medicine, Cleveland, OH 44106, USA; 3 Department of Ophthalmology, Massachusetts Eye and Ear, Harvard Medical School, Boston, MA 02115, USA; 4 Department of Ophthalmology, Duke University, Durham, NC 27705, USA

**Keywords:** glaucoma, polygenic risk score, genetic risk score, diversity, glaucoma genetics

## Abstract

Glaucoma is the leading cause of irreversible blindness worldwide. Primary open-angle glaucoma (POAG), the most common glaucoma subtype, is more prevalent and severe in individuals of African ancestry. Unfortunately, this ancestral group has been historically under-represented among genetic studies of POAG. Moreover, both genetic and polygenic risk scores (GRS, PRS) that are typically based on genetic data from European-descent populations are not transferable to individuals without a majority of European ancestry. Given the aspirations of leveraging genetic information for precision medicine, GRS and PRS demonstrate clinical potential but fall short, in part due to the lack of diversity in these studies. Prioritizing diversity in the discovery of risk variants will improve the performance and utility of GRS and PRS-derived risk estimation for disease stratification, which could bring about earlier POAG intervention and treatment for a disease that often goes undetected until significant damage has occurred.

## 1. Introduction

Glaucoma is the leading cause of irreversible blindness worldwide [1]; primary open-angle glaucoma (POAG) is the most common subtype. POAG is a degenerative optic nerve disease characterized by progressive peripheral vision loss that results in complete and irreversible blindness without early diagnosis and treatment. POAG is a complex, multifactorial disease, for which genetic factors are one of the most prominent culprits, given the high heritability (ranging from 0.17–0.81 [2,3]). Others have extensively reviewed the current state of the glaucoma genetic literature [4,5]. We focus herein on genetic and polygenic risk scores as well as the importance of diversity in polygenic risk of POAG.

## 2. Genetic and Polygenic Risk Scores

Genome-wide association studies (GWAS) have identified associations between hundreds of thousands of potential loci and thousands of phenotypes, with many loci conferring very small effect sizes [6], consequently limiting clinical applications of individual variants. Therefore, a need has emerged to aggregate the effects of individual risk loci into a cumulative risk estimate. In recent years, genetic risk scores (GRS) and polygenic risk scores (PRS) have become an area of focus [7]. GRS and PRS are similar in that they combine effects at associated loci into a single measure. GRS commonly include variants that show a statistically significant association at the genome-wide level. PRS aggregate genome-wide variants and additional risk variants spanning the genome to capture the additive effects of genome-wide variation. PRS cast a wider net and have shown promise for predicting the genetic risk of complex diseases [8] including breast cancer [9], cardiovascular [10], and Alzheimer’s [11] diseases. As clinical translational tools, GRS and PRS could expedite the attainment of precision medicine [12]. With further refinement, clinicians may be able to better estimate patient risk for disease and identify appropriate personalized prevention or disease management measures [13].

Glaucoma is a disease for which early screening is beneficial; due to the high heritability of glaucoma, a goal in the field is to expand early detection methods to include screening via genetics. In glaucoma, variation in *MYOC* can cause early-onset glaucoma. Indeed, the clinical utility of screening for POAG-causing *MYOC* variants has been shown [14] though in a study design not necessarily reflective of a typical clinical setting. Furthermore, *MYOC*-driven glaucoma is one of the few that occurs early in life and where variation in a single gene can have a significant effect. By contrast, most genetic variants that influence glaucoma arise from combined factors or interactions that would have a negligible effect if presented singularly. Growing evidence indicates the likely future utility of GRS and PRS in POAG prediction and classification [15,16]. Despite crucial yet incremental steps toward clinical utility, the broad application of GRS/PRS remains limited by many factors, including the fact that POAG risk variants identified through GWAS are not all causative, functional variants. Therefore, the future implementation of GRS and PRS in POAG hinges on the discovery of more genetic loci. Furthermore, while individual genetic screening can be performed via DNA sequencing, the diagnostic yield remains low (20%) [17]. Generalized genetic screening for POAG may be useful for those over the age of 50, as recent studies have shown an area under the receiver operating characteristic curve score of 0.76 [17]. However, barriers to this type of genetic testing include the following: (i) individual variants often have a small effect compared to sociodemographic or environmental factors, (ii) the sensitivity and specificity of these tests do not align with cutoffs for cost-effectiveness, and (iii) each proposed test needs additional validation in a clinical setting. When considering screening for populations younger than 50 years of age, more genetic risk factors will need to be discovered to propose and implement a clinically useful model. Importantly, and as the main focus of this overview, the genetic diversity in these studies is sparse, limiting the generalizability to other ancestral groups. While expanding diversity is a key priority in the field, an exact timeline for a predictive tool cannot be established at this time.

## 3. Limited Cross-Ancestry Application of Genetic and Polygenic Risk Scores

Upwards of 80% of GWAS were performed in populations of predominantly European ancestry [12,18,19]. In the construction of GRS and PRS, the use of base data (GWAS results) from European-descent populations typically leads to worse predictive ability in diverse (non-majority European ancestry) populations. This likely reflects the different effects that variants may have and the different allele frequencies at which those variants are present in geographically separated and/or isolated ancestral groups [12,19,20,21]. The predictive value of European-derived PRS for complex phenotypes such as lipid traits, cardiometabolic diseases (coronary artery disease and type 2 diabetes), and cancer is significantly diminished in diverse populations (e.g., [20,21,22,23,24]). PRS performance improves when risk variants discovered in diverse ancestral groups are included [19]. Therefore, the next logical step to improve PRS performance in diverse groups is to expand the diversity of samples in the underlying GWAS analyses.

In an early attempt to emphasize the need for diverse representation in POAG genetic studies, Liu and colleagues evaluated POAG-associated variants discovered in European-descent individuals in a sample of majority African ancestry individuals [25]. Unsurprisingly, the 57 single nucleotide polymorphisms (SNPs) including well-established SNPs in *CDKN2B-AS1, TMCO1, CAV1/CAV2*, chromosome 8q22 intergenic region, and *SIX1/SIX6* were not as significantly associated with POAG in a majority African-descent samples. It logically follows that applying a GRS based on the majority of existing literature would yield a risk-screening tool that is not universally applicable across diverse ancestral groups.

## 4. Representation of Populations of African Descent in Primary Open-Angle Glaucoma Studies

Historically, POAG GWAS have predominantly been performed in samples with an overwhelming majority of European ancestry [26]. Unsurprisingly, most POAG loci identified in this way demonstrate a stronger association with POAG risk in European-descent than African-descent individuals (e.g., [27,28]). Given the variability in allele frequency, linkage disequilibrium (LD), and underlying genetic architecture between ancestral populations, limited cross-ancestry variation is to be expected [29]. Others have consistently shown that the failure of known POAG loci to replicate at genome-wide significance in POAG GWAS of African ancestry populations [27,28,30,31,32,33] results, in part because of reduced statistical power due to admixture. Population diversity in Africa raises similar challenges to genetic analysis [34]. These findings likely reflect real differences in the genetic architecture of POAG among populations with global African genetic ancestry.

In the first glaucoma GWAS and admixture analysis in African Americans, Hoffman and colleagues detected an association between African ancestry and glaucoma incidence in African Americans in the Women’s Health Initiative (WHI) [30]. Furthermore, known POAG variants failed to replicate. A GRS of 10 POAG lead variants identified in European-descent GWAS was only nominally significant in African American prevalent cases.

The first POAG GWAS performed in continental African participants, the Genetics in Glaucoma patients of African descent (GIGA) study, validated *TXNRD2, CDKN2B-AS1*, and *TMCO1* loci previously reported to confer POAG risk in Europeans [31]. Additionally, they identified a novel association between POAG and *EXOC4*, a ubiquitously expressed gene involved in exocytosis. Replication of this effect was not achieved due to the rarity of this variant in other continental African populations, for which small sample sizes were available. To investigate GRS performance across different ancestries, they tested how POAG risk variants identified from European and Asian populations fared in estimating risk in an African cohort. The GRS, comprised of variants for which allele frequencies varied greatly between the groups, was significantly associated with POAG but only explained four per cent of the variance after adjustment.

The African Descent and Glaucoma Evaluation Study (ADAGES) III GWAS detected a putative novel association with advanced glaucoma at *EN04* [35]. Association or trending association was observed at known European ancestry-associated loci, including: 9p21 (*CDKN2BAS*), *FNDC3B*, *8q22*, *AFAP1*, and *TMCO1*. However, LD and conditional analyses indicated potential independence from previously reported SNPs. A 400-SNP PRS based on these results had an area under the receiver operating characteristic curve of 0.94 and performed significantly better than GRS based on European-descent POAG loci. This is not surprising given that base and test data overlapped, a practice that is generally avoided in contemporary GRS and PRS analyses.

The unique genetic architecture of POAG in African-descent populations is also supported by the discovery of a POAG locus unique to individuals of African ancestry in the Genetics of Glaucoma in People of African Descent (GGLAD) Consortium [32]. GGLAD identified a novel POAG risk locus at amyloid-β A4 precursor protein-binding family B member 2 (*APBB2*), an association that was replicated in multiple independent datasets. Importantly, while the rs59892895*C risk allele was present at an appreciable minor allele frequency in African ancestry populations, it was less than 0.1% in individuals of European or Asian ancestry. It would follow, then, that ancestry is a major key to understanding genetic risk for POAG among African and African-descent samples, and there are African genetic signals that have yet to be detected. Furthermore, a subset of known POAG loci (26 variants identified in individuals of European and Asian ancestry) was evaluated in the GGLAD study via inverse-variance, fixed-effects meta-analysis. Odd ratios (OR) for 23 of 26 variants were smaller in African than European ancestry individuals, demonstrating both the utility of African-descent discovery analyses and the lack of transferability between populations for a subset of POAG loci identified in studies of European and Asian ancestry groups.

The Primary Open-Angle African American Glaucoma Genetics (POAAGG) study performed GWAS and admixture mapping in their cohort. Significant differences in case-control ancestry were detected, and a 23-SNP GRS was significant, providing evidence that known POAG SNPs together with omni-genic ancestry effects influence POAG risk [27,36].

**Figure 1:**
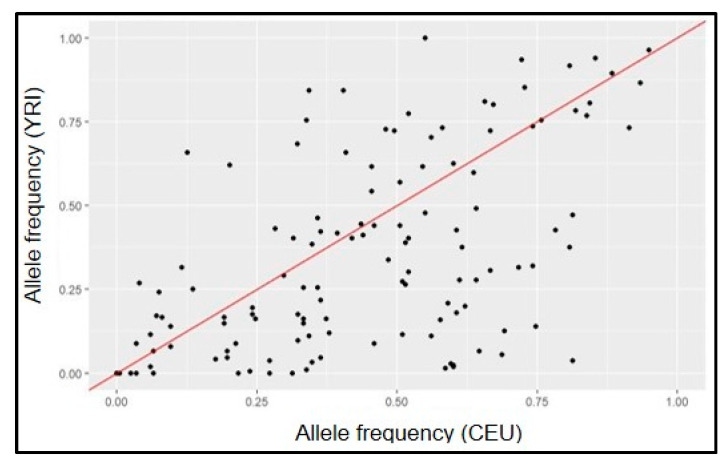
Comparison of allele frequencies of 127 known POAG SNPs in ancestral populations (1000G YRI vs. CEU).

Importantly, more recent large-scale, global efforts to elucidate the genetic architecture of POAG have included samples from populations of diverse ancestry. The most contemporary, which included the GGLAD and other African-ancestry focused studies in the largest to-date trans-ancestry glaucoma genetics study by the International Glaucoma Genetics Consortium (IGGC) identified 127 POAG loci [28]. African-specific subset analyses identified a locus in *IQGAP1* that failed to replicate in samples of majority European and Asian ancestry. While this work represents a major global effort, it is important to note that because more than 98% of the samples were of majority European and Asian descent, African-specific signals are likely drowned out. It is additionally important to acknowledge that this trans-ancestry study reported 127 variants, some of which are vastly different effects across different ancestries. Figure 1 shows that allele frequencies for many of the 127 most recently identified POAG loci differ between European-descent (CEU) and African (YRI) ancestral populations as represented by 1000 Genomes (Phase 3) data [37].

We recently showed that a GRS built on this most current set of POAG risk variants consistently underperforms in African Americans [33]. Expanding diverse representation in PRS development and testing represents an opportunity for increased understanding of POAG genetic risk and facilitate a path for precision medicine for those at risk for POAG.

## 5. Clinical Applicability of Genetic Data in POAG Risk Stratification and Intervention Relies on Transferability to Ensure Reduction of Health Disparities

Growing evidence indicates utility of GRS and PRS in POAG prediction and classification [4,15,16], yet most is shown with data from mostly individuals of European ancestry. GRS have not yet been established as a clinical screening tool for POAG. Neustaeter and colleagues are testing feasibility of POAG GRS for clinical screening using a prospective approach [38], but the variants included in the GRS were identified from POAG GWAS in European-descent individuals. In European-descent samples, a higher burden of genetic risk captured by PRS is associated with POAG age at diagnosis and disease-relevant clinical parameters [15,16]. However, in African-descent groups the predictive value diminishes [33]. This is not surprising, as current POAG PRS fail to incorporate genetic ancestry, significantly dampening transferability across ancestral groups [29].

Importantly, when evaluated in aggregate via GRS, known POAG variants effectively classify people of European descent who are at the highest genetic risk for POAG. We [39] and others [4,16,40,41,42] have shown varying degrees of applicability for GRS and/or PRS to target POAG screening and initiate timely therapy. Risk scores of 12–1200 variants associated with intraocular pressure (IOP) and/or POAG have shown an association with increased POAG risk in case-control analyses. In case-only analyses, these variants are associated with earlier age at diagnosis, more family members with POAG, need for incisional surgery, maximum recorded IOP, and more severe disease [16,39,40,41,42,43]. We previously showed that a 12-variant GRS is associated with increased POAG risk. Each higher GRS unit is associated with 0.36-year earlier POAG diagnosis [43] and mean age at diagnosis was 5.2 years earlier in the top vs. bottom 5% of the GRS distribution. The most recent report examining PRS in POAG showed that high glaucoma polygenic risk was associated with a more rapid rate of glaucomatous visual field worsening and a faster rate of nerve fiber layer thinning [4]. Siggs and colleagues showed that, despite early detection, some patients with POAG experience rapid disease onset and vision loss. They also demonstrated the benefit of informative PRS in clinical detection of severe disease risk. Individuals with high polygenic risk, defined as those in the top 5% of a normal distribution, were 50% more likely to experience vision loss compared to the bottom 95%, despite this group receiving more intense treatment. Notably, none of the studies have been replicated in people of African descent, thus representing an important gap in the field of POAG genetics.

To this end, we recently evaluated the 127 known POAG variants via GRS in the Million Veteran Program (MVP) Biobank, taking into account ancestry-specific and meta-analysis effect sizes from the IGGC [33]. While the GRS were significantly associated with POAG in European Americans and African Americans in the MVP, a higher proportion of people of European descent were consistently categorized in the top GRS decile when compared to people of African descent (21.9–23.6% and 12.9–14.5%, respectively). We further found that GRS was significantly associated with the documentation of invasive glaucoma surgery in European-descent cases; however, this association was significant in African-descent cases only if the GRS was weighted by ancestry-specific effect estimates. While unsurprising, these data further emphasize the need for understanding POAG genomic discovery in African-descent populations.

## 6. Beyond Genetics: A Note on Prioritizing Diversity in Ophthalmology

As efforts are being made to increase diversity of genetic data, so too are efforts needed to expand the diversity of clinical ophthalmological data. Such efforts are recently underway. Addis and colleagues call for the expansion of standard, normalized clinical databases containing clinical ocular data [44]. In evaluating the Cirrus High-Definition Optical Coherence Tomography database, they found that clinical measures differed significantly when comparing the normative database to POAAGG controls for disc area, rim area, cup-to-disc ratio, and cup volume. This study is another call to diversity in “standard” or “normalized” databases used for diagnosing diseases. Since we are aware of these racial and ethnic discrepancies in the presentation and progression of glaucoma, a one-size-fits-all diagnostic device may not be sufficient to catch all glaucoma-affected people, especially those of minoritized groups. As such efforts expand, care must be taken to clinically incorporate appropriate measures of ancestral diversity rather than apply race-based measures or modifiers.

## 7. Global Perspectives

Recently, Soh and colleagues reported that globally, more than half of all glaucoma cases were undetected [45]. Africa and Asia had higher odds of undetected glaucoma when compared to Europe. Asia is projected to have the most cases of undetected glaucoma due to population size alone, while Africa is projected to have the highest increase in undetected cases. Countries with lower human development indices (HDI) had higher proportions of undetected glaucoma when compared to countries with medium to high HDIs. However, the percentage of undetected glaucoma in high HDI groups (70%) is still concerning because glaucoma is often asymptomatic until the late stages of the disease. A large proportion of undetected glaucoma cases presented with at least a moderate visual field deficit. The blindness due to glaucoma is irreversible, and it is vital that screening and outreach efforts be proportionate to the amount of risk in each country. Tailored interventions need to be implemented in high-risk populations as well as underserved or resource-poor areas. Timely, cost-effective glaucoma screening is crucial due to the asymptomatic nature of the disease and the cost of vision care. It follows that a majority of those living with glaucoma are undiagnosed, and therefore, untreated. The accessibility of vision care needs to be more critically assessed due to the associations between poor visual function and worse quality of life [46].

Early POAG intervention preserves vision [47]; to intervene early in high-risk groups including African-descent populations, wherein POAG presents earlier [48] and progresses faster, current screening approaches must be updated. If genetics are expected to contribute to early detection, which is highly likely given the high heritability of POAG, then it is crucial to [26,49,50] increase African and African-descent representation in POAG studies that identify genetic loci influencing POAG risk. Prioritizing African-descent POAG in genetics is logical and necessary to attain precision ocular health in historically excluded and high-risk populations. Care should also be taken to acknowledge different recruitment strategies and study design efforts that go beyond Eurocentric models, including partnering with Black leaders to ensure collaboration and active participation in research [51]. Without this intentional focus on increasing diversity in POAG research, the disparity between those at highest risk and those receiving appropriate preventative care will likely widen.

## 8. Summary

The lack of diversity in POAG genetics research is consistent with GWAS of many complex diseases, which have historically been performed using overwhelmingly Eurocentric data [6,29]. These studies have consequently lacked sufficient data to enable extrapolation to populations that have historically been underrepresented in research. This dearth of data could limit the reach of the benefits of personalized medicine and the genomics revolution in underrepresented populations. This is especially true with diseases such as glaucoma, where both the prevalence and severity of disease is greater in African-descent populations, which have been historically underrepresented in genomics studies. When addressing diseases characterized by such dramatic health disparities, it is imperative that GRS and PRS include data from all populations, especially those at most risk for the disease. Inclusion of diverse data in genomic discovery studies increases the likelihood that novel risk variants will be identified. In the long-term, the glaucoma genetics literature suggests that improving glaucoma risk modeling may make early intervention possible, which could ensure better health outcomes. These advancements in risk stratification and intervention would help make the visions of precision medicine and health equity more realistic.

## Data Availability

Not applicable.
